# Comparing the Volatile and Soluble Profiles of Fermented and Integrated Chinese Bayberry Wine with HS-SPME GC–MS and UHPLC Q-TOF

**DOI:** 10.3390/foods12071546

**Published:** 2023-04-06

**Authors:** Yingjie Miao, Gaowei Hu, Xiaolong Sun, Yashi Li, Huanting Huang, Yongqian Fu

**Affiliations:** Taizhou Key Laboratory of Biomass Functional Materials Development and Application/Zhejiang Provincial Key Laboratory of Plant Evolutionary Ecology and Conservation, School of Life Sciences, Taizhou University, Taizhou 318000, China

**Keywords:** Chinese bayberry, fermented bayberry wine, integrated bayberry wine, HS-SPME GC–MS, UHPLC Q-TOF

## Abstract

To evaluate the flavor characteristics of Chinese bayberry alcoholic beverages, fermented bayberry wine (FBW) and integrated bayberry wine (IBW) were investigated for their volatile and soluble profiles using HS-SPME GC–MS and UHPLC Q-TOF and were analyzed with multidimensional statistical analysis, including PCA and OPLS-DA. The volatile compounds 1-pentanol, β-caryophyllene and isopentanol were only detected in IBW. β-caryophyllene, the key flavor component of bayberry, was found to be the most abundant volatile compound in IBW (25.89%) and was 3.73 times more abundant in IBW than in FBW. The levels of ethyl octanoate, ethyl nonanoate, and ethyl decanoate were also several times higher in IBW than in FBW. These compounds contributed to the strong bayberry aroma and better fruity flavor of IBW. On the other hand, high levels of ethyl acetate and octanoic acid in FBW, representing pineapple/overripe or sweat odor, were key contributors to the fermented flavor of FBW. Soluble sugars, such as sucrose, D-glucose, and D-tagatose, as well as amino acids, such as L-glutamate and L-aspartate, had much higher levels in IBW. The anthocyanin pigment cyanidin 3-glucoside, which generates red color, was also higher in IBW. On the other hand, most of the differentially expressed alcohols, acids, amino acids, purines/pyrimidines and esters were present in higher concentrations in FBW compared to IBW. This demonstrated that IBW has a much sweeter and more savory taste as well as a better color generated by more anthocyanins, while FBW presents a more acidic and drier taste as well as a complex formation of alcohols and esters. The study also prompts the need for further research on the flavor profiles of IBW and its potential application and market value.

## 1. Introduction

Chinese bayberry (*Myrica rubra* Sieb. and Zucc.) is an important fruit tree widely planted in the east and south regions of China [1]. Its fruits have an attractive color, a unique flavor, and are known for their various biological benefits [2,3,4,5]. The tree matures from June to July, with the ripening period lasting only about 30 days. The fruit is highly prone to damage from mechanical forces, and its postharvest shelf life is limited to just two days at 20 °C or less than 7 days at 4 °C [6], making it challenging for long-term storage and transportation [7]. To deal with a large quantity of fruit ripening in a short period and to extend the consumption time, additional processing is often necessary. The main products made from Chinese bayberry fruit include dried fruit, preserved fruit, canned fruit, fruit juice, and alcoholic beverages [8,9].

There are typically two methods for making alcoholic beverages from Chinese bayberry. The first is to produce fermented bayberry wine (FBW) by fermenting the fruit must or juice with or without yeast inoculation [10]. The second method is to create a blended alcoholic beverage, known as integrated bayberry wine (IBW), by either incorporating whole fruits or adding fruit juice to crude fermented wine, distilled alcoholic spirits, or edible alcohol. Due to the substantial differences in the brewing processes of fermented wine and integrated wine, which can involve very different technical approaches, it is likely that their flavor profiles will vary significantly.

There have been numerous studies on the determination of fruit wine flavors [11]. Fermented fruit wine obtains its flavors from the materials used, such as fruit juice or whole fruits, as well as the fermentation process [12]. The final aroma and flavor profile of a fermented fruit wine is heavily influenced by all aspects of pre-fermentative treatments, the fermentation process, and post-fermentation treatments, including yeast strains, the fermentation process, and maturation strategies [13]. In general, fermentation-derived volatiles make up the majority of the total aroma composition of wine [11]. On the other hand, the wine production process extracts color and flavor components from fruit-derived materials. For example, the color of red wines comes from anthocyanin pigments found in the skins of dark-colored grapes [14]. However, to the best of our knowledge, there have been few studies on the flavor profiles and qualities of IBW. Thus, it was deemed necessary to evaluate the flavor characteristics of IBW and compare them to FBW.

The objective of the current study was to identify the distinct aroma and flavor profiles, as well as the physical and chemical properties, of IBW and FBW. Additionally, the study aimed to examine the impact of brewing methods on the taste of the alcoholic beverage and to investigate the possible reasons and mechanisms behind the differences. The findings of this study can provide valuable information for the development of new brewing methods, as well as the optimization of existing ones. The results of the study can also provide a better understanding of the factors and the potential corresponding mechanisms that influence the aroma and flavor profiles of fruit wine.

## 2. Materials and Methods

### 2.1. Bayberry Harvesting and Juice Extraction

Undamaged mature-stage Chinese bayberry cultivar Dongkui was carefully harvested from an orchard in Xianju, Zhejiang province, China. The fruits were promptly transported to the laboratory within 4 h and stored at 4 °C during sorting and analysis and at −20 °C for later use. Before use, the bayberries were thawed at room temperature and their pits were removed through hole punching. The fruit was then homogenized using a juice extractor to obtain bayberry juice, which was filtered using a four-layer gauze, sterilized at 85 °C for 15 min, and cooled to room temperature. The fresh Chinese bayberry juice had a total soluble solid content of 12°Brix, total sugar content of 101 g/L, titratable acidity of 10.3 g/L expressed as citric acid, and a pH of 2.90.

### 2.2. Fermented Bayberry Wine

Three kilograms of fresh bayberry juice was transferred into a 4 L sterile glass cylindrical container. Sucrose was added to reach a total soluble solid content of 22°Brix. A calculated amount of 50 mg/L sulfur dioxide was then provided by adding potassium metabisulfite. A commercial yeast strain of *S. cerevisiae* RW was inoculated at a rate of 0.2% (*m*/*v*). The containers were kept at a temperature of 20 °C in the absence of light. After 10–15 days, the alcoholic fermentation was completed (when the total sugar loss was less than 0.5 g/L per day), and the wine was pressed through a stainless-steel sieve. Another calculated amount of 50 mg/L sulfur dioxide was added, and the wine was settled in 1 L glass carboys and cooled at 4 °C for 72 h. No malolactic fermentation was conducted. The wine was stored at 17 °C and analyzed within 1 month.

### 2.3. Integrated Bayberry Wine

Equal volumes of bayberry juice and 25% (*v*/*v*) edible alcohol were mixed together to make IBW. The mixture was transferred into a 4 L sterile glass cylindrical container and preserved with a calculated amount of 50 mg/L sulfur dioxide. The containers were stored at 20 °C, away from light, for two weeks. Afterward, the wine was filtered through a stainless-steel sieve, stabilized with a calculated amount of 50 mg/L sulfur dioxide, allowed to settle in 1 L glass carboys, and refrigerated at 4 °C for 72 h. The wine was then stored at 17 °C and analyzed within two weeks.

### 2.4. Total Soluble Solids, pH, and Titratable Acidity

The measurement of total soluble solids (TSS) was performed using a digital refractometer and was expressed in °Brix. The pH was measured with a FE20K pH meter (Mettler Toledo, Columbus, OH, USA). Titratable acidity (TA) was measured by titration with 0.1 mol/L NaOH and was expressed as the percentage of citric acid [15].

### 2.5. Volatile Compounds Analyzed by HS-SPME GC–MS

Five milliliters of wine was placed in a 20 mL vial which was then sealed using Teflon/silicone. The vial was equilibrated at 45 °C for 25 min. The volatiles were extracted using a headspace solid-phase microextraction (HS-SPME) device with a Supelco 50/30 μm, DVB/CAR/PDMS fiber (Supelco Inc., Bellefonte, PA, USA). The headspace of the vial was extracted at 45 °C for 40 min, after which the fiber was introduced to the injector and desorbed at 220 °C for two mins.

The volatiles were then analyzed using a GC–MS QP2010 plus (Shimadzu, Kyoto, Japan) gas chromatography–mass spectrometer system with a VOCOL capillary column (60 m × 0.32 mm × 1.8 μm) (Supelco Inc., Bellefonte, PA, USA). The carrier gas used was helium, and the oven temperature program started at 35 °C for three minutes and was finally ramped to 210 °C and maintained for 15 min. The temperatures of the ion source and the analyzer were set at 200 °C and 210 °C, respectively. The detector voltage was operated at 0.9 kV and the mass range from *m*/*z* 45 to 600 was scanned to generate characteristic peaks. Overlapping peaks were separated using the GC–MS Solution Workstation software Ver. 4. Six sample replicates were performed in each group.

Under the same chromatography conditions as for the sample, 1 μL n-alkane mixed standard (C8–C20, chromatographically pure, Sigma Aldrich, St. Louis, MO, USA) was used for HS-SPME GC–MS. The retention time (Rt) of each alkane was determined, and the equation to calculate the linear retention index (RI) of unknown components is as follows:

RI(x) = 100 × n + 100 × [Rt(x) − Rt(n)]/[Rt(n + 1) − Rt(n)].

The identification of volatile compounds was carried out by comparing the peaks of mass spectra to those in the NIST and Wiley libraries. The quantities of volatiles were calculated based on their peak areas and expressed as the relative content, which is the percentage of the total chromatographic area.

### 2.6. Soluble Components Analyzed by UHPLC Q-TOF

#### 2.6.1. Preparation of HPLC

After thawing slowly at 4 °C, 100 μL samples were dissolved in 400 μL pre-cooled methanol/acetonitrile solution (1:1, *v*/*v*), mixed, and placed at −20 °C for 30 min, then centrifuged at 14,000 g at 4 °C for 20 min. The supernatant was obtained and dried in a vacuum before samples were dissolved in 100 μL acetonitrile aqueous solution (acetonitrile: water = 1:1, *v*/*v*) and centrifuged at 14,000 g 4 °C for 15 min; then the supernatant was analyzed.

#### 2.6.2. Ultra-High-Performance Liquid Chromatography

Samples were separated on an Agilent 1290 Infinity LC Ultra-High-Performance Liquid Chromatography System (UHPLC) with a Waters ACQUITY UPLC BEH Amide 1.7 µm, 2.1 mm × 100 mm column. The column temperature was 25 °C; the flow rate was 0.3 mL/min; and the injection volume was 2 μL. Mobile phase composition A (MA): water + 25 mM ammonium acetate + 25 mM ammonia; mobile phase composition B (MB): acetonitrile. The gradient elution procedure was as follows: 0–1 min, 95% MB; 1–14 min, MB varied linearly from 95% to 65%; 14–16 min, MB linearly changed from 65% to 40%; 16–18 min, MB was maintained at 40%; 18–18.1 min, MB varied linearly from 40% to 95%; 18.1–23 min, MB was maintained at 95%. The sample was placed in a 4 °C autosampler throughout the analysis. To avoid the effects of instrumental detection signal fluctuations, continuous analysis of samples was performed in a random order. QC samples were inserted into the sample queue to monitor and evaluate the stability of the system and the reliability of the experimental data.

#### 2.6.3. Q-TOF Mass Spectrometry

The sample was separated by UHPLC and subjected to a Triple TOF 5600 mass spectrometer (AB SCIEX). positive and negative ion modes of electrospray ionization (ESI) were used for detection. The settings of the ESI source were as follows: Ion Source Gas1 (Gas1): 60; Ion Source Gas2 (Gas2): 60; curtain gas (CUR): 30; source temperature: 600 °C; IonSapary Voltage Floating (ISVF): ±5500 V (positive and negative modes); TOF MS scan m/z range: 60–1000 Da; product ion scan m/z range: 25–1000 Da; TOF MS scan accumulation time: 0.20 s/spectra; product ion scan accumulation time: 0.05 s/spectra. The secondary mass spectra were obtained using information-dependent acquisition (IDA) with high-sensitivity mode, a declustering potential (DP) of ±60 V (positive and negative modes), collision energy of 35 ± 15 eV, and IDA settings were as follows: exclude isotopes within 4 Da, candidate ions to monitor per cycle: 6. Six sample replicates were performed in each group.

The raw data were converted to mzXML format by ProteoWizard, and then the XCMS program was used for peak alignment, retention time correction, and peak area calculation. Metabolite structures were retrieved in a self-built database and identified using a method combining accurate primary mass matching (<25 ppm) and secondary spectra matching. For the data extracted by the XCMS, ion peaks with >50% missing values in the group were deleted.

### 2.7. Statistical Analysis

SIMCA-P 14.1 (Umetrics, Umea, Sweden) was used for pattern recognition. The data were preprocessed using Pareto scaling for multidimensional statistical analysis, including unsupervised principal component analysis (PCA) and orthogonal partial least-squares discriminant analysis (OPLS-DA). Single-dimensional statistical analysis included Student’s *t*-test and analysis of multiple sources of variation; volcano plots were drawn using R software.

## 3. Results and Discussion

### 3.1. Total Soluble Solids, pH, and Titratable Acidity

Bayberry juice was obtained through homogenization and filtration, followed by sterilization. The total soluble solids (TSS), pH, and titratable acidity (TA) of the juice were measured immediately after sterilization and cooling. The ethanol content, residual sugar (RS), TA, and dry extract of the wine were determined after wine production was completed. These results are shown in Table 1.

The flavor of IBW is largely determined by its raw material composition and recipe. Besides fresh bayberries and a base wine such as crude fermented wine, distilled alcoholic spirit, or edible alcohol, additional ingredients such as a color stabilizer, an acidity regulator, and preservatives are typically required. However, to minimize experimental interference, only fresh bayberries and base wine were used in this study.

For FBW, the residual sugar mainly originated from bayberry juice and the sucrose added during processing, and its concentration was significantly lower than that in IBW. Additionally, the ethanol content in the FBW was much lower than that of the IBW, which is probably the greatest difference regarding the general composition between the two wines. For IBW, residual sugar mainly comes from the bayberry juice, with a small amount coming from the base wine. In this study, edible alcohol was used as the base wine so as to minimize the impact of the base wine on the flavor of wine. The RS in the IBW was slightly lower than the theoretical value, calculated based on the sugar concentration in the juice. On the other hand, the ethanol content in the IBW was slightly higher than the theoretical value, calculated based on the initial concentration of edible alcohol. This was likely due to the less strict pasteurization method used in this study. The surviving yeast underwent mild fermentation during the juice processing and wine preparation process, resulting in the conversion of part of the sugar into ethanol in the IBW.

### 3.2. Volatile Compounds of FBW and IBW

Volatile compounds in FBW and IBW were extracted using a HS-SPME device and analyzed through GC–MS QP2010 and with a VOCOL capillary column. Table 2 shows that a total of 41 volatile compounds were detected in 12 wine samples of both groups, including 9 alcohols, 4 aldehydes, 7 acids, 13 esters, 2 terpenes, and 6 other compounds. The odor descriptions of volatile compounds were obtained from the Flavornet website (http://www.flavornet.org/ accessed on 30 October 2022) and the literature [1,10,16,17,18].

In the case of IBW samples, 36 volatile compounds from various chemical classes were identified, including 8 alcohols (28.76%), 3 aldehydes (2.00%), 7 acids (6.02%), 11 esters (31.94%), 1 terpene (25.89%), and 6 others (5.39%). The most abundant compound was caryophyllene, accounting for approximately 25.89% of the total GC peak area, followed by 2-phenylethyl alcohol (11.20%) and ethyl acetate (11.19%).

In the case of FBW samples, 35 volatile compounds from various chemical classes were identified, including 7 alcohols (37.81%), 3 aldehydes (2.24%), 6 acids (2.65%), 12 esters (33.49%), 2 terpenes (7.42%), and 5 others (16.39%). The most abundant compound was ethyl acetate, accounting for around 20.18% of the total GC peak area, followed by 2-phenylethyl alcohol (17.70%) and 3,3-dimethyl-1,2-epoxybutane (12.03%).

It is worth noting that some compounds in the “others” category, particularly 3,3,4,4-tetrafluoro-1,5-hexadiene, might have originated from consumable material and experimental operations as contaminants instead of being natural volatile compounds of wine. Therefore, most of these compounds were excluded from further analysis and discussion.

### 3.3. PCA and OPLS-DA Analysis of Volatile Compounds

**Figure 1:**
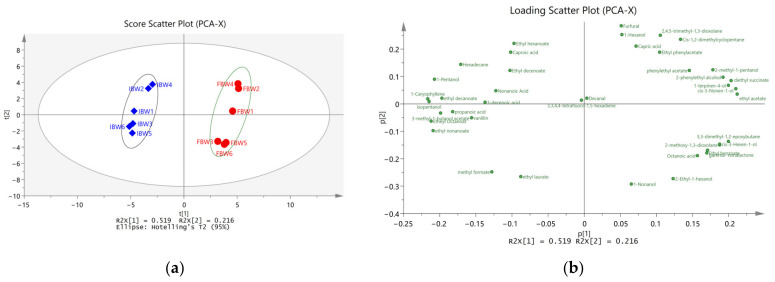
The PCA score plot and loading plot of volatile compounds: (**a**) Scores scatter plot t1 vs. t2; t[1] and t[2] represent PC1 and PC2. (**b**) Loadings scatter plot p1 vs. p2.

**Figure 2:**
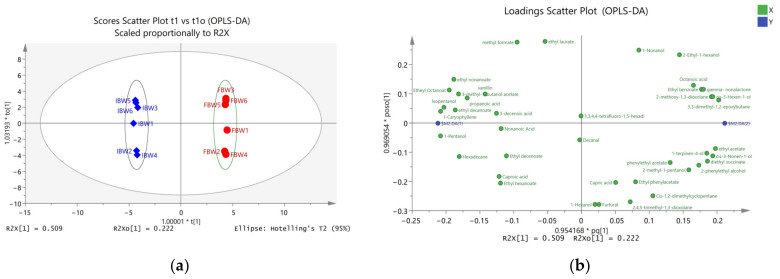
The OPLS-DA score plot and loading plot of volatile compounds: (**a**) Scores scatter plot t1 vs. t1o; (**b**) loadings scatter plot pq1 vs. pos1o.

SIMCA 14.1 was employed to carry out multidimensional statistical analysis. The results of unsupervised PCA and supervised OPLS-DA analysis are shown in Figure 1 and Figure 2.

Principal component analysis (PCA) is an unsupervised method of data analysis that combines the original set of compounds into a new set of comprehensive variables known as principal components (PCs). The goal of PCA is to reduce the dimension of the data by selecting several PCs that reflect as much information as possible about the original variables. Figure 1a displays the score scatter plot of the first two principal components (PC1 and PC2), showing the differences and similarities between IBW and FBW samples. Figure 1b is the loadings plot, illustrating the correlation between volatile compounds and their relative importance in contributing to the differences or similarities observed in the scatter plot.

As shown in Figure 1, the first two PCs (PC1 and PC2) account for 73.5% of the total contribution of the original dataset. The plot reveals that the 12 wine samples are grouped into two clusters, marked with an ellipse. The IBW samples form one cluster on the left side (represented by blue diamonds), while the FBW samples form another cluster on the right side (represented by red circles). The clear distinction between the two groups indicates that the model provides good discrimination.

Orthogonal partial least-squares discriminant analysis (OPLS-DA) is a supervised statistical method distinct from principal component analysis (PCA). Unlike PCA, OPLS-DA uses partial least-squares regression to model the relationship between the concentration of compounds and sample categories, and to discriminate samples into multiple categories. In the OPLS-DA score plot, there are two principal components: the predictive principal component and the orthogonal principal component. The plot only has one predictive principal component, t1, while there can be multiple orthogonal principal components. OPLS-DA maximizes the differences between groups on t1, thus allowing for a direct distinction of inter-group variations on t1, while it reflects the intra-group variations on the orthogonal principal components.

Figure 2 displays the OPLS-DA score and loading plots of soluble compounds. The 12 wine samples are clearly clustered into two groups, marked with ellipses, and colored with blue diamonds for IBW samples and red circles for FBW samples. A clear distinction between the two groups indicates a successful discrimination of the model. The variable importance in projection (VIP) is calculated to measure the contribution and explanatory ability of the concentrations of each compound in the classification and discrimination of samples. This information is used to assist in the screening of marker compounds, with the screening standard being VIP > 1.0 and *p* < 0.05. A total of 21 volatile compounds with significant difference (VSD) were screened and are shown in Table 3.

According to the VIP values, the VSDs that made the greatest contribution to the differences between IBW and FBW were 1-pentanol, β-caryophyllene, and isopentanol. As shown in Table 3, compounds such as 1-pentanol (balsamic odor), isopentanol (whiskey, malt, burnt odor), hexadecane (alkane odor), and 3-methyl-1-butanol acetate (banana odor) were only detected in IBW. Conversely, cis-3-hexen-1-ol (green odor), 1-terpinen-4-ol (woody, turpentine, nutmeg odor), γ-nonalactone (coconut, peach odor), and ethyl benzoate (chamomile, floral, celery, fruit odor) were only detected in FBW. Among the VSDs, propanoic acid showed the highest fold change of 18.13 (IBW/FBW), followed by 2-methoxy-1,3-dioxolane with a fold change of 16.25 (FBW/IBW) and octanoic acid with a fold change of 10.97 (FBW/IBW).

The high content of aldehydes and terpenes is one of the most important characteristics of the volatile components in bayberry. Specially, β-caryophyllene has been identified as the most dominant terpene compound and the key flavor component of bayberry in previous studies [1,16,18,19]. Our results show that β-caryophyllene was the most abundant volatile compound in IBW and was 3.73 times more abundant in IBW than in FBW. This high concentration of β-caryophyllene (25.89%) contributed highly to the strong bayberry flavor of IBW compared to FBW. However, the number of terpenoids and aldehydes was significantly less than the results reported in the above studies. This demonstrated that most terpenoids and aldehydes from bayberry juice were not retained in the final wine, but were degraded or volatilized during the brewing process.

It has been reported that certain characteristic aroma volatiles in bayberry, such as 3-hexen-1-ol (green), 3-nonen-1-ol (cucumber), and terpinen-4-ol (wood, turpentine, nutmeg), significantly increased after fermentation in bayberry juice [17]. Our study confirms these findings, as 3-hexen-1-ol, 3-nonen-1-ol, and terpinen-4-ol were much higher in FBW than in IBW. Interestingly, while cis-3-hexen-1-ol, which represents a fresh and green note, was not detected in IBW, it was present in FBW. Further studies are needed to validate this result and reveal possible underlying mechanisms. However, our results showed a different trend compared to a previous study, as 1-pentanol and isopentanol were only detected in IBW. 1-Pentanol is described as having a balsamic flavor and fusel oil taste; it contributes to the taste and aroma of wines by balancing the flavor profile and improving the overall sensory experience of wine. It has been reported that the content of 1-pentanol gradually increased during the storage of NFC Chinese bayberry juice [18]. Isopentanol, a common higher alcohol in wine, can add a fruity, floral note to the flavor of wine in small amounts, and may exhibit unpleasant off-flavors at higher concentrations. However, due to the relatively low concentration of 1-pentanol and isopentanol, they should have little negative impact on the sensory properties of IBW.

High levels of ethyl acetate and octanoic acid were an important characteristic of FBW. Ethyl acetate representing a pineapple or overripe flavor is a key contributor to the aroma of fermented alcoholic beverages and bayberry wine. On the other hand, the levels of ethyl octanoate (fruit and fat flavor), ethyl nonanoate (fruit and flower flavor), and ethyl decanoate (grape flavor) were more than two times higher in IBW than in FBW, contributing to the better fruity flavor of IBW compared to FBW.

Interestingly, it was observed that the non-flavor compound 3, 3-dimethyl-1, 2-epoxybutane was present at a higher level in FBW, 7.45 times higher than that in IBW. This compound has been reported to be produced by bacteria and exhibits strong inhibitory activity against *Aspergillus flavus* [20]. The higher concentration in FBW might indicate that similar microorganisms were present during bayberry wine fermentation. Further investigation is needed to fully understand the mechanism behind the generation of this compound.

### 3.4. Soluble Compounds of FBW and IBW

**Figure 3:**
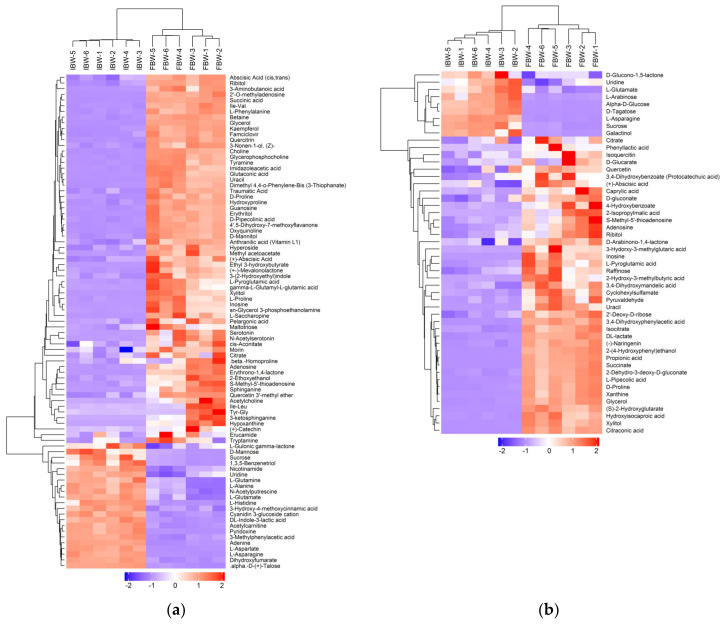
Hierarchical clustering plot of soluble compounds detected by electrospray ionization (ESI), with positive and negative ion modes. (**a**) Detected by positive ion mode; (**b**) detected by negative ion mode.

The composition of the soluble compounds in FBW and IBW was analyzed using UHPLC Q-TOF. A total of 5227 and 2808 peaks were detected in positive and negative ion modes, respectively, with 87 and 50 compounds being identified. Hierarchical clustering was performed on the qualitative data to visualize the relationship between samples and the differences in quantitative levels of compounds, as shown in Figure 3.

**Figure 4:**
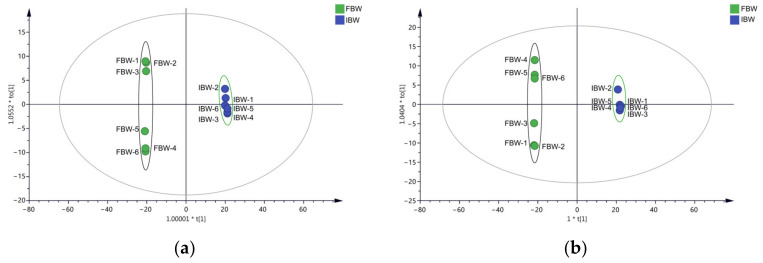
The OPLS-DA score plots of soluble compounds detected by electrospray ionization (ESI), with positive and negative ion modes. (**a**) Detected by positive ion mode; (**b**) detected by negative ion mode.

The soluble compounds detected in the positive and negative ion modes were analyzed using OPLS-DA to establish the correlation between the concentration of the compounds and sample categories, and to differentiate samples into several groups. The OPLS-DA score and loading plots of the soluble compounds are depicted in Figure 4. The 12 wine samples are clearly separated into two groups, the IBW (represented by blue circles) and FBW (represented by green circles) groups, in both positive and negative ion modes. The clear separation between the two groups shows the effectiveness of the discrimination model.

### 3.5. OPLS-DA Analysis of Soluble Compounds

After multidimensional statistical analysis of the soluble compounds detected by both positive and negative ion modes, compounds with VIP > 1, *p* < 0.01, and FC > 2 or <0.5 were selected as soluble compounds with significant difference (SSD). All SSDs are listed in Table 4, consisting of 6 sugars, 10 alcohols, 16 acids, 9 amino acids, 6 purines and pyrimidines, 7 esters, and 15 other compounds.

Among the differentially expressed sugars, D-glucose had the highest VIP value of 14.70, followed by sucrose with a value of 12.22 and D-tagatose with 7.27. D-tagatose also had the highest fold change, with a ratio of 95.46 (IBW/FBW), followed by Alpha-D-glucose at 59.36 and sucrose at 12.75. Sucrose, D-glucose, and D-tagatose can be considered as the most important differentially expressed sugars as they are the main contributors to the sweetness of bayberry juice. During fermentation, yeast typically metabolizes sugars to produce alcohol and acids; thus, most carbohydrate substances in IBW are higher in concentration compared to those in FBW, with the exception of raffinose.

The differential expression of alcohols was analyzed, with more than half of them being sugar alcohol derivatives such as galactinol, xylitol, and ribitol. Galactinol had the highest VIP (11.92), while 3-ketosphinganine had the highest fold change value (FBW/IBW = 163.26). Most of the differentially expressed alcohols in FBW were present in higher concentrations compared to IBW, and it is believed that they were produced through yeast fermentation. Sphinganine and 3-ketosphinganine, found in high concentrations in FBW, are metabolites involved in the synthesis and metabolic pathway of sphingomyelin, a major structural lipid in eukaryotic membranes, and could have been produced and released by yeast during fermentation.

All differentially expressed acids in FBW showed several times- to several hundred times-higher concentrations than those in IBW, with no exceptions. 2-Isopropylmalic acid had the highest fold change (FBW/IBW = 351.38), followed by (S)-2-hydroxyglutarate (FBW/IBW = 60.91) and citraconic acid (FBW/IBW = 48.55). As acids in fermented wine are mainly transformed from sugars during fermentation, which barely occurs in IBW, the higher concentration of acids in FBW is an important sensory characteristic, but less so in IBW. In general, appropriate concentrations of volatile acids are ideal for producing high-quality wines, and a deacidification treatment could help to improve the flavor quality of FBW [21]. In addition, more esters would be formed through the esterification of alcohols with organic acids during the fermentation, post-fermentation, and aging processes, which is confirmed by the higher levels of esters in FBW.

In addition, the differential expression of amino acids was also analyzed. L-Saccharopine had the highest fold change (FBW/IBW = 43.07), followed by Ile-Val (FBW/IBW = 18.45) and 3-aminobutanoic acid (FBW/IBW = 12.10). L-Saccharopine (an intermediate compound in the metabolic pathway of L-lysine), Ile-Val (a dipeptide consisting of two essential amino acids isoleucine and valine), and 3-aminobutanoic acid had higher concentrations in FBW than in IBW. On the other hand, L-glutamate and L-aspartate, which contribute to a savory or umami taste in foods, had higher concentrations in IBW than in FBW. Although these compounds do not have a strong or distinctive taste or flavor, they provide specific contributions to the final wine flavor.

The concentration of purines/pyrimidines, esters, and other compounds in FBW were mostly higher than in IBW, with only a few exceptions. For instance, the level of anthocyanin pigment cyanidin 3-glucoside, which generates red color, was higher in IBW. 1,3,5-Benzenetriol, an odorless and tasteless phenolic compound commonly found in wine, had a concentration in IBW that was 11.93 times higher than that in FBW. However, the exact role of 1,3,5-benzenetriol in contributing to bayberry taste remains unclear and further research is required to determine its effect on flavor and aroma. Additionally, the anthocyanin pigment cyanidin 3-glucoside cation, which gives bayberry fruits their red or purple color, had a concentration in IBW that was 2.92 times higher than that in FBW.

## 4. Conclusions

To evaluate the flavor characteristics of Chinese bayberry alcoholic beverages, fermented bayberry wine (FBW) and integrated bayberry wine (IBW) were investigated for their volatile and soluble profiles using HS-SPME GC–MS and UHPLC Q-TOF and analyzed with multidimensional statistical analysis, including PCA and OPLS-DA.

The volatile compounds 1-pentanol, 1-caryophyllene, and isopentanol were only detected in IBW. β-caryophyllene, the key flavor component of bayberry, was found to be the most abundant volatile compound in IBW (25.89%), and was 3.73 times more abundant in IBW than in FBW. The levels of ethyl octanoate, ethyl nonanoate, and ethyl decanoate were also several times higher in IBW than in FBW. These compounds contributed to the strong bayberry aroma and better fruity flavor of IBW. On the other hand, high levels of ethyl acetate and octanoic acid in FBW, representing pineapple/overripe or sweat odor, were key contributors to the fermented flavor of FBW.

Soluble sugars, such as sucrose, D-glucose, and D-tagatose, as well as amino acids, such as L-glutamate and L-aspartate, had much higher levels in IBW. The anthocyanin pigment cyanidin 3-glucoside, which generates red color, was also higher in concentration in IBW. On the other hand, most of the differentially expressed alcohols, acids, amino acids, purines/pyrimidines, and esters were present in higher concentrations in FBW compared to IBW. This demonstrated that IBW has a much sweeter and more savory taste, as well as a better color generated by more anthocyanins, while FBW presents a more acidic and drier taste, as well as a complex formation of alcohols and esters.

Further efforts are needed to deepen our understanding of the difference between IBW and FBW due to the limitations of our instrumental environment and experimental experience. The study also prompts the need for further research on the flavor profiles of IBW and its potential application and market value.

## Figures and Tables

**Table 1:** General composition of Chinese bayberry wines.

	Composition of Wine			
	Ethanol(%)	RS (g/L)	TA (%)	Dry Extract (g/L)
IBW	12.81 ± 0.41 ^a^	4.92 ± 0.06 ^a^	1.44 ± 0.11 ^a^	20.48 ± 0.31 ^a^
FBW	10.51 ± 0.18 ^b^	3.81 ± 0.18 ^b^	1.27 ± 0.06 ^b^	19.31 ± 0.44 ^b^

TSS, Total soluble solids; TA, titratable acidity; RS, residual sugar; data with significant differences (*p* < 0.05, *t*-test) in each row are represented by different superscript letters.

**Table 2:** Relative abundance of volatile compounds from Chinese bayberry wine.

	Volatile Compounds	Odor Description	RI	Relative Abundance (%)	
				IBW	FBW
Alcohols	1-pentanol	balsamic	825	3.82 ± 0.58	nd*
	isopentanol	whiskey, malt, burnt	829	3.58 ± 0.79	nd
	2-methyl-1-pentanol		861	4.09 ± 1.84	7.24 ± 1.24
	cis-3-hexen-1-ol	green	966	nd	0.72 ± 0.25
	1-hexanol	resin, flower, green	969	1.07 ± 0.52	1.14 ± 0.24
	2-ethyl-1-hexanol	rose, green	1097	2.02 ± 1.01	3.79 ± 1.07
	cis-3-nonen-1-ol	cucumber	1228	1.63 ± 1.11	5.16 ± 0.45
	1-nonanol	fat, green	1232	1.36 ± 0.58	2.07 ± 1.12
	2-phenylethyl alcohol	honey, spice, rose, lilac	1245	11.20 ± 2.06	17.70 ± 2.91
Aldehydes	furfural	bread, almond, sweet	1176	0.43 ± 0.26	0.49 ± 0.30
	decanal	soap, orange peel, tallow	1280	0.46 ± 0.51	0.45 ± 0.29
	γ -nonalactone	coconut, peach	1531	nd	1.30 ± 0.63
	vanillin	vanilla	1596	1.12 ± 0.97	nd
Acids	propanoic acid	pungent, rancid, soy	<800	0.91 ± 0.51	0.05 ± 0.08
	3-methyl-4-oxopentanoic acid		<800	3.35 ± 0.78	0.86 ± 0.36
	caproic acid	sweat	1045	0.27 ± 0.29	nd
	octanoic acid	sweat, cheese	1220	0.11 ± 0.14	1.17 ± 0.64
	nonanoic acid	green, fat	1330	0.56 ± 0.50	0.08 ± 0.19
	3-decenoic acid		1375	0.63 ± 0.34	0.15 ± 0.36
	capric acid		1443	0.21 ± 0.32	0.35 ± 0.34
Esters	ethyl acetate	pineapple, overripe	<800	11.19 ± 1.77	20.18 ± 1.96
	methyl formate		<800	2.18 ± 0.72	1.55 ± 0.65
	3-methyl-1-butanol acetate	banana	974	0.79 ± 0.36	nd
	ethyl hexanoate	apple peel, fruit	1073	1.46 ± 0.94	0.53 ± 0.48
	etheyl octanoate	fruit, fat	1255	4.49 ± 0.61	1.57 ± 0.72
	diethyl succinate	wine, fruit	1272	1.56 ± 0.37	3.73 ± 0.83
	ethyl benzoate	chamomile, flower, celery, fruit	1292	nd	0.87 ± 0.44
	ethyl nonanoate	fruit, flower	1367	2.08 ± 0.36	0.78 ± 0.37
	ethyl decenoate		1375	0.24 ± 0.24	0.04 ± 0.09
	ethyl phenylacetate	fruit, sweet	1387	0.12 ± 0.20	0.29 ± 0.26
	phenylethyl acetate	fruit, sweet, rose	1402	nd	0.20 ± 0.19
	ethyl decanoate	grape	1473	5.94 ± 1.43	2.14 ± 0.65
	ethyl laurate	leaf	1637	1.89 ± 0.30	1.63 ± 0.67
Terpenes	1-terpinen-4-ol	wood, turpentine, nutmeg	1283	nd	0.49 ± 0.21
	β-caryophyllene	wood, spice	1547	25.89 ± 2.59	6.93 ± 1.38
others	3,3,4,4-tetrafluoro-1,5-hexadiene		812	1.89 ± 0.77	1.89 ± 0.71
	2-methoxy-1,3-dioxolane		844	0.05 ± 0.13	0.87 ± 0.27
	2,4,5-trimethyl-1,3-dioxolane		850	1.04 ± 0.31	1.31 ± 0.48
	3,3-dimethyl-1,2-epoxybutane		857	1.62 ± 0.74	12.03 ± 2.37
	cis-1,2-dimethylcyclopentane		1234	0.06 ± 0.09	0.30 ± 0.31
	hexadecane		1600	0.73 ± 0.34	nd

* nd, not detected.

**Table 3:** Volatile compounds screened out by variable importance in projection (VIP) value of OPLS-DA analysis.

Volatile Compounds	Odor Description	VIP	FC(IBW/FBW)	FC(FBW/IBW)
1-pentanol	balsamic	1.22924	∞ *	0
β-caryophyllene	wood, spice	1.22741	3.73	0.27
isopentanol	whiskey, malt, burnt	1.20884	∞	0
3,3-dimethyl-1,2-epoxybutane		1.20727	0.13	7.45
ethyl acetate	pineapple, overripe	1.17866	0.55	1.80
ethyl octanoate	fruit, fat	1.1774	2.87	0.35
cis-3-nonen-1-ol	cucumber	1.17538	0.32	3.16
cis-3-hexen-1-ol	green	1.17054	0	∞
ethyl nonanoate	fruit, flower	1.15655	2.67	0.37
2-methoxy-1,3-dioxolane		1.15386	0.06	16.25
ethyl decanoate	grape	1.13974	2.78	0.36
1-terpinen-4-ol	wood, turpentine, nutmeg	1.13623	0	∞
diethyl succinate	wine, fruit	1.13137	0.42	2.39
hexadecane		1.12445	∞	0
3-methyl-1-butanol acetate	banana	1.12155	∞	0
γ -nonalactone	coconut, peach	1.11397	0	∞
ethyl benzoate	chamomile, flower, celery, fruit	1.10583	0	∞
2-phenylethyl alcohol	honey, spice, rose, lilac	1.07205	0.63	1.58
octanoic acid	sweat, cheese	1.05611	0.09	10.97
propanoic acid	pungent, rancid, soy	1.03633	18.13	0.06
2-methyl-1-pentanol		1.00204	0.56	1.77

* ∞, not detected in the other group of samples. FC, fold change.

**Table 4:** Soluble compounds screened by variable importance in projection (VIP > 1.5), fold change (FC > 2 or FC < 0.5), and *p* value (*p* < 0.01) of OPLS-DA analysis.

	Peak Name	*m*/*z* *	rt(s)	Adduct	Description	Ion Mode	VIP	Fold Change	
								(IBW/FBW)	(FBW/IBW)
Sugars	M503T799	503.16	798.85	(M-H)^−^	Raffinose	NEG	1.80	0.43	2.32
	M163T171	163.06	170.53	(M+H-H_2_O)^+^	D-(+)-Talose	POS	5.37	4.07	0.25
	M198T701	198.10	700.95	(M+NH_4_)^+^	D-Mannose	POS	2.77	9.98	0.10
	M341T701	341.11	701.41	(M-H)^−^	Sucrose	NEG	12.22	12.75	0.08
	M179T499	179.06	498.97	(M-H)^−^	D-Glucose	NEG	14.70	59.36	0.02
	M179T559	179.06	558.83	(M-H)^−^	D-Tagatose	NEG	7.27	95.46	0.01
Alcohols	M300T71_1	300.29	70.72	(M+H)^+^	3-ketosphinganine	POS	6.55	0.01	163.26
	M302T248	302.30	248.49	(M+H)^+^	Sphinganine	POS	3.81	0.01	92.93
	M137T68_2	137.06	68.40	(M-H)^−^	2-(4-Hydroxyphenyl)ethanol	NEG	5.28	0.02	57.83
	M151T449	151.06	449.18	(M-H)^−^	Xylitol	NEG	1.78	0.03	38.53
	M110T192	110.08	192.28	(M+NH_4_)^+^	Glycerol	POS	1.52	0.08	13.03
	M203T56	203.16	56.31	(M+CH_3_COO+2H)^+^	(Z)-3-Nonen-1-ol,	POS	2.09	0.09	11.10
	M183T568	183.09	568.05	(M+H)^+^	D-Mannitol	POS	1.57	0.25	4.08
	M194T119	194.10	118.67	(M+CH_3_CN+H)^+^	Ribitol	POS	1.83	0.43	2.30
	M401T702	401.13	702.43	(M+CH_3_COO)^−^	Galactinol	NEG	11.92	27.51	0.04
	M170T200	170.08	199.94	(M+H)^+^	Pyridoxine	POS	2.17	13.29	0.08
Acids	M175T554	175.06	554.46	(M-H)^−^	2-Isopropylmalic acid	NEG	3.85	0.00	351.38
	M147T753_2	147.03	752.87	(M-H)^−^	(S)-2-Hydroxyglutarate	NEG	5.10	0.02	60.91
	M129T356_2	129.02	356.20	(M-H)^−^	Citraconic acid	NEG	4.01	0.02	48.55
	M117T736	117.02	735.65	(M-H)-	Succinate	NEG	6.67	0.03	28.74
	M131T759	131.03	759.02	(M+H)^+^	Glutaconic acid	POS	1.66	0.04	27.92
	M73T736	73.03	735.75	(M-H)^−^	Propionic acid	NEG	1.83	0.04	27.28
	M131T230	131.07	229.95	(M-H)^−^	Hydroxyisocaproic acid	NEG	2.91	0.04	25.38
	M167T73	167.03	72.78	(M-H)^−^	3,4-Dihydroxyphenylacetic acid	NEG	2.52	0.09	11.51
	M89T424	89.02	423.59	(M-H)^−^	DL-lactate	NEG	3.06	0.11	9.42
	M117T273	117.06	273.43	(M-H)^−^	2-Hydroxy-3-methylbutyric acid	NEG	1.55	0.11	8.96
	M178T121	178.05	120.63	(M-H)^−^	Cyclohexylsulfamate	NEG	2.60	0.13	7.60
	M173T696	173.01	696.38	(M-H_2_O-H)^−^	Isocitrate	NEG	1.51	0.25	3.94
	M153T443	153.02	442.94	(M-H)^−^	3,4-Dihydroxybenzoate (Protocatechuic acid)	NEG	1.84	0.29	3.48
	M130T578	130.09	577.87	(M+H)^+^	D-Pipecolinic acid	POS	1.97	0.30	3.29
	M282T158	282.17	157.55	(M+NH_4_)^+^	Abscisic Acid (cis, trans)	POS	2.08	0.31	3.23
	M263T144	263.13	144.14	(M-H)^−^	(+)-Abscisic acid	NEG	2.39	0.38	2.66
Amino Acids	M277T870	277.14	869.79	(M+H)^+^	L-Saccharopine	POS	2.02	0.02	43.07
	M231T71	231.17	70.60	(M+H)^+^	Ile-Val	POS	2.41	0.05	18.45
	M104T96	104.07	95.71	(M+H)^+^	3-Aminobutanoic acid	POS	2.34	0.08	12.10
	M114T579	114.06	578.85	(M-H)^−^	D-Proline	NEG	2.40	0.21	4.69
	M166T474	166.09	474.49	(M+H)^+^	L-Phenylalanine	POS	1.68	0.22	4.51
	M130T569	130.05	569.13	(M+H)^+^	L-Pyroglutamic acid	POS	2.97	0.34	2.97
	M148T764	148.06	763.66	(M+H)^+^	L-Glutamate	POS	2.15	2.34	0.43
	M133T715	133.06	714.69	(M+H)^+^	L-Asparagine	POS	2.49	9.47	0.11
	M134T773	134.04	773.14	(M+H)^+^	L-Aspartate	POS	1.59	9.61	0.10
Purines/pyrimidines	M151T389	151.03	389.12	(M-H)^−^	Xanthine	NEG	2.23	0.01	136.75
	M111T150	111.02	149.91	(M-H)^−^	Uracil	NEG	4.68	0.01	76.95
	M282T191	282.12	191.44	(M+H)^+^	2′-O-methyladenosine	POS	4.45	0.02	63.31
	M137T306	137.04	305.82	(M+H)^+^	Hypoxanthine	POS	2.29	0.05	18.39
	M268T312	268.10	312.36	(M+H)^+^	Adenosine	POS	4.48	0.35	2.88
	M136T290_2	136.06	290.47	(M+H)^+^	Adenine	POS	3.52	10.98	0.09
Esters	M115T60_2	115.07	60.07	(M+H-H_2_O)^+^	Ethyl 3-hydroxybutyrate	POS	2.84	0.02	43.84
	M101T243	101.02	243.28	(M+H-H_2_O)^+^	Erythrono-1,4-lactone	POS	2.35	0.04	23.41
	M117T78	117.05	78.45	(M+H)^+^	Methyl acetoacetate	POS	1.53	0.07	13.76
	M343T58	343.05	58.15	(M+H)^+^	Dimethyl 4,4-o-Phenylene-Bis (3-Thiophanate)	POS	1.69	0.10	10.43
	M207T123	207.05	123.33	(M+CH_3_COO)^−^	D-Arabinono-1,4-lactone	NEG	2.78	0.50	2.01
	M177T190	177.04	189.63	(M-H)^−^	D-Glucono-1,5-lactone	NEG	2.37	1.94	0.51
	M149T295	149.08	295.48	(M+H)^+^	Dihydroxyfumarate	POS	1.56	2.49	0.40
Others	M146T350	146.12	350.27	(M+H)^+^	Acetylcholine	POS	2.64	0.02	54.78
	M120T60	120.08	60.03	(M+H-H_2_O)^+^	Tyramine	POS	1.83	0.02	41.18
	M449T302	449.11	301.56	(M+H)^+^	Quercitrin	POS	5.28	0.12	8.07
	M287T79	287.09	79.20	(M+H)^+^	4′,5-Dihydroxy-7-methoxyflavanone	POS	1.56	0.15	6.53
	M271T67	271.06	66.52	(M-H)^−^	(-)-Naringenin	NEG	2.07	0.23	4.37
	M317T128	317.07	127.75	(M+H)^+^	Quercetin 3′-methyl ether	POS	1.64	0.24	4.23
	M287T302	287.05	302.01	(M+H)^+^	Kaempferol	POS	2.05	0.24	4.20
	M258T736	258.11	735.67	M^+^	Glycerophosphocholine	POS	5.52	0.25	3.93
	M298T150	298.10	149.98	(M+H)^+^	S-Methyl-5′-thioadenosine	POS	6.10	0.26	3.78
	M118T510_2	118.09	510.29	(M+H)^+^	Betaine	POS	4.71	0.28	3.57
	M104T494	104.11	494.32	M^+^	Choline	POS	10.66	0.35	2.90
	M268T259	268.14	258.63	(M+CH_3_CN+Na)^+^	Acetylcarnitine	POS	1.71	1.93	0.52
	M123T93	123.05	93.02	(M+H)^+^	Nicotinamide	POS	2.01	2.15	0.47
	M449T479	449.11	479.35	M^+^	Cyanidin 3-glucoside	POS	3.82	2.92	0.34
	M127T703	127.04	702.88	(M+H)^+^	1,3,5-Benzenetriol	POS	2.28	11.93	0.08

* *m*/*z*: mass-to-charge ratio, rt(s): Retention time (s).

## Data Availability

The datasets generated and/or analyzed during the current study are available from the corresponding author on reasonable request.
